# Responses of the plant community characteristics and diversity of abandoned grasslands in the Loess Hilly Region in China to slope aspect and year of abandonment

**DOI:** 10.3389/fpls.2026.1754831

**Published:** 2026-03-30

**Authors:** Liping Li, Haonan He, Jinghong Pei, Xiaoxi Zhang, Meiyan Fang, Qirui Ye, Yiwei Feng, Shujie Xi, Jiangwen Li

**Affiliations:** 1 College of Life Sciences, Yan’an University, Yan’an, China; 2 Shaanxi Engineering and Technological Research Center for Conservation and Utilization of Regional Biological Resources, Yan’an University, Yan’an, China; 3 Pratacultural Engineering Research Center of Yan’an City, Yan’an, China

**Keywords:** loess hilly region, plant functional biodiversity, plant species composition, plant structural biodiversity, slope aspect, years of abandonment

## Abstract

**Introduction:**

As key temporal and topographical factors, the year of abandonment and slope aspect significantly affect the restoration process and plant community assembly of abandoned grasslands by altering their microenvironmental conditions. However, how their interactive effects specifically influence the spatiotemporal variations in quantitative characteristics, functional groups, and species or functional diversity of grassland communities remains unclear.

**Methods:**

In the present study, abandoned grasslands in the Loess Hilly Region, China, were taken as the research objects. The response patterns of the quantitative characteristics of the grassland community, species belonging to different functional groups, and the species or functional diversity of the grassland community to years of abandonment, slope aspect and their interactions were investigated.

**Results:**

Slope aspect and year of abandonment had significant interactive effects on the quantitative community characteristics, species of each functional group, and species/functional diversity of grasslands. In particular, the dynamic patterns of the above indicators with increasing years of abandonment revealed significant slope aspect heterogeneity. With increasing years of abandonment, all the quantitative characteristics of the shady slope communities tended to continuously decease, or first increase but then decrease, and the species number, biomass, density of each functional group, and community-level species and functional diversity presented similar changes. In contrast, the corresponding indicators of sunny slope communities were dominated by no significant change or an overall increasing trend.

**Conclusion:**

In general, the overall traits of the abandoned grassland communities on shady slopes tended to improve rapidly, followed by a decline during the succession process, whereas those on sunny slopes tended to be stable or showed overall positive development. To manage the ecological restoration process of abandoned grasslands, different strategies need to be formulated on the basis of slope aspect heterogeneity.

## 1 Introduction

The Loess Plateau in China is among the regions that have experienced the most severe soil erosion worldwide (Bai et al., 2024). In recent decades, the implementation of a series of major ecological projects has led to the formation of large areas of abandoned grassland in this region. Understanding the spatiotemporal changes in the composition and the diversity in both species and functions of the plant species in the aforementioned abandoned grassland communities is highly important for guiding the subsequent restoration of the local ecological environment. According to previous studies, abandonment usually causes changes in soil nutrient status, such as increases in soil organic matter and total and available nitrogen contents (Jiao et al., 2013); however, on a spatial scale, severe water erosion affects various topographies (Rao et al., 2024) and thus causes the redistribution of water and nutrients in abandoned grassland (Zhao et al., 2025). These two factors lead to dynamic alterations in local ecosystems, which directly cause high instability in the species composition of the grassland community (Qiao et al., 2018; Sără、eanu et al., 2023). In contrast, this instability might lead to nutrient imbalances between the soil and plants. These changes might result in shifts in species composition and alter the dominance of plant functional groups with distinct ecological strategies (Puyang et al., 2021). Therefore, in-depth exploration of the comprehensive influences of abandonment and microtopography on grassland communities in this eco-fragile region is highly important.

Previous studies have indicated that with increasing years of abandonment, the content of soil nutrients increases (Heydari et al., 2020), and annual and biennial species are usually replaced by perennial semishrub herbs or even shrubs, which are dominated by Fabaceae Lindl., Asteraceae Bercht. & J. Presl and Poaceae Barnh. plants (Shi et al., 2023; Sun et al., 2017). In addition to structural complexity, the species diversity of grassland communities also increases (Guo et al., 2017). However, microtopographic factors can also change environmental conditions, such as light and soil moisture conditions, by affecting spatial heterogeneity (Cen et al., 2022) and consequently leading to alterations in the species composition of plant communities (Guo et al., 2017). Slope aspect is among the most important microtopographic factors influencing the succession of grassland communities and significantly affects their structure, function and species diversity (Khan et al., 2023), as it usually significantly affects microenvironmental conditions, such as light and soil moisture, and the adaptability of plants with different functional traits. In general, sunny slopes usually have relatively high temperatures and low soil moisture contents, which negatively affect the accumulation of organic matter (Qin et al., 2016). These factors might provide favorable soil temperature conditions to support the growth of various plant species, resulting in increased species diversity; however, in some cases, these conditions might also result in low diversity, as relatively arid, low-temperature and infertile soil conditions limit the survival of some occasionally shade-tolerant species (Guo Q. et al., 2025). Therefore, the effects of years of abandonment on these functional groups of plants might be quite different in grasslands with different slopes, leading to various alteration patterns in the species composition and diversity of plant communities (Vannucchi et al., 2022). However, whether the aforementioned phenomena are bound to occur and the underlying mechanisms are still unclear.

Therefore, grasslands with different years of abandonment in the Loess Hilly Region in China were taken as objects. On the basis of vegetation investigations of the grasslands on shady and sunny slopes, the effects of changes in both aspects and years of abandonment on the grassland community were studied. These results may provide a scientific basis for understanding the spatiotemporal dynamics of grassland communities and their main drivers in this region and thus provide scientific recommendations for promoting the sustainable development of local grassland ecosystems. We hypothesized that the environmental differences between shady and sunny slopes may result in distinct trajectories of community quantitative characteristics, functional groups, species and functional diversity with increasing years of abandonment.

## 2 Materials and methods

### 2.1 Study region

The study region is located in the Miaozuigou watershed (36°37′-36°40′ N, 109°21′-109°23′ E) of Yan’an city, China, which belongs to the Loess Hilly Region of the Loess Plateau, with an altitude of 1123~1243 m. The climate here is classified as a warm temperate semiarid continental monsoon climate, with an average annual temperature of 7.8 °C, an average annual active accumulated temperature of 3446.7 °C, an average annual precipitation of 478.3 mm, an average annual sunshine duration of 2300~2700 h, and a frost-free duration of 96~146 d. The soil here is classified as a Calcic Cambisol according to the American Soil Taxonomy. In the abandoned regions here, the dominant species in the grassland are *Setaria viridis* (Linn.) Beauv., *Heteropappus altaicus* (Willd.) Novopokr., *Patrinia heterophylla* Bunge, *Dendranthema indicum* (Linn.) Des Moul., *Dracocephalum moldavica* Linn., *Poa annua* Linn., *Phragmites australis* (Cav.) Trin. ex Steud., *Stipa capillata* Linn., *Artemisia gmelinii* Web.et Stechm., *Artemisia capillaris* Thunb., *Sophora davidii* (Franch.) Skeels, etc (Zhang et al., 2025). Apart from the artificially planted shrublands, the grassland with the longest abandonment time in the local area is still in the late stage of grassland succession and is composed mainly of perennial semishrub herbaceous plants.

### 2.2 Vegetation investigation and sampling

Four grasslands with similar site conditions (**Table 1**) but different abandoned stages according to their years of abandonment were selected (initial, abandoned for < 10 yr), middle (10-20 yr), mid-late (20-30 yr) and late (>30 yr) stages. When we selected the grassland, we confirmed that the plots had similar planting and abandonment histories and no human disturbances through farmer interviews and government records, which controlled the impact of historical contingency to the greatest extent. Because of the difficulties in determining the specific years of abandonment of different grasslands in the study region, to ensure the comparability of the data, only one plot with an area over 600 m^2^ was established on the shady and sunny slopes of each grassland, and only four grasslands were used. A similar plot setup method was also used in the study of Baldi et al. (2025). Six fixed quadrats (replicates) were subsequently randomly established in each plot, resulting in the formation of a total of 48 quadrats (four abandonment years × two slope aspects × six replicates). In accordance with previous vegetation investigations using the nested quadrat method, the minimum representative area of the community in each sampling plot was less than 1 m²; thus, the area of every quadrat was set as 1 m × 1 m.

**Table 1:** Information on the studied sample plots.

Years of abandonment (yr)	Geographical coordinates	Elevation (m a.s.l)	Slope aspect	Dominant species of the community
<10	109°21′38″E, 36°38′50″N	1133	Sunny slope	*A. capillaris*
			Shady slope	*S. viridis*
10-20	109°21′38″E, 36°38′42″N	1136	Sunny slope	*H. altaicus*
			Shady slope	*C. indicum*
20-30	109°21′37″E, 36°38′42″N	1126	Sunny slope	*P. australis*
			Shady slope	*S. grandis*
>30	109°21′31″E, 36°38′55″N	1180	Sunny slope	*A. gmelinii*
			Shady slope	*S. davidii*

Information on all plant species recorded in every plot in this study is provided in **Supplementary Table 1**.

A vegetation investigation was conducted in the aforementioned fixed quadrats in August, and all the recorded plant species are listed in the supplementary file (**Supplementary Table 1**).

The plants observed were distinguished by their photosynthetic pathway, reproduction mode, root type and growth form according to the *Flora of China* (Editorial Committee of Flora of China, 1979). By doing so, the plants were distinguished into C3 and C4 plants, sexual reproducing (SR) and sexual and asexual reproducing (S&AR) plants, taproot and fibrous root plants (TR and FR plants) and perennial forbs (PF), perennial grasses (PG), annual and biennial herbs (ABH) and shrub and semishrub herbs (S&SH). The species identity, average height, coverage and density of the plants in every quadrat were subsequently recorded. All the plants in the fixed quadrants were subsequently cut, and their aboveground parts were subsequently oven-dried at 65 °C for 24 h to a constant weight to determine the biomass of the whole community or the groups with given functional traits (Jia et al., 2024).

### 2.3 Data processing and statistical analysis

The α diversity indices, including the species richness index, Shannon–Wiener index, evenness index and dominance index, were calculated according to the methods of Pan et al. (2023) to assess the level of community organization. In accordance with our unpublished data on the functional traits of the observed plant species, functional diversity indices, including functional richness (FRic), functional evenness (FEve) and functional dispersion (FDis) indices, were calculated using FDiversity software (Casanoves et al., 2011).

Generalized linear mixed models (GLMMs) were used to examine the effects of fixed factors (slope aspect, abandonment year, and their interaction) on all the indices referred to in Section 2.2. The quadrat ID nested within plot ID was included as a random intercept to control the spatial heterogeneity among the quadrats. Because only one plot was established for each combination of abandonment year and slope aspect due to field constraints in the study area, as aforementioned in Section 2.2, the effects of plot ID were confounded with those of abandonment and slope aspect. Therefore, plot effects were not included as random effects. Where applicable, following significant fixed effects, Tukey’s honestly significant difference (HSD) test was conducted for pairwise *post hoc* comparisons. All GLMM analyses were performed using IBM SPSS 29.0 software, with the significance level set at α = 0.05 for all the statistical tests. OriginPro 2021b software was used for figure preparation.

## 3 Results

### 3.1 Response of grassland community quantitative characteristics to slope aspect and years of abandonment

A generalized linear mixed model (GLMM) analysis revealed that slope aspect, abandonment year, and their interaction significantly affected the average height, coverage, density, and biomass of the grassland community (*P* < 0.05; **Supplementary Table 2**). In addition, although the random effects of quadrat were significant, its proportion of its explanation for model variation for every community quantitative trait was quite low (0.48~0.76%, *P* < 0.05; **Supplementary Table 2**). Therefore, in the subsequent analysis, the influence of fixed effects is mainly considered.

**Figure 1:**
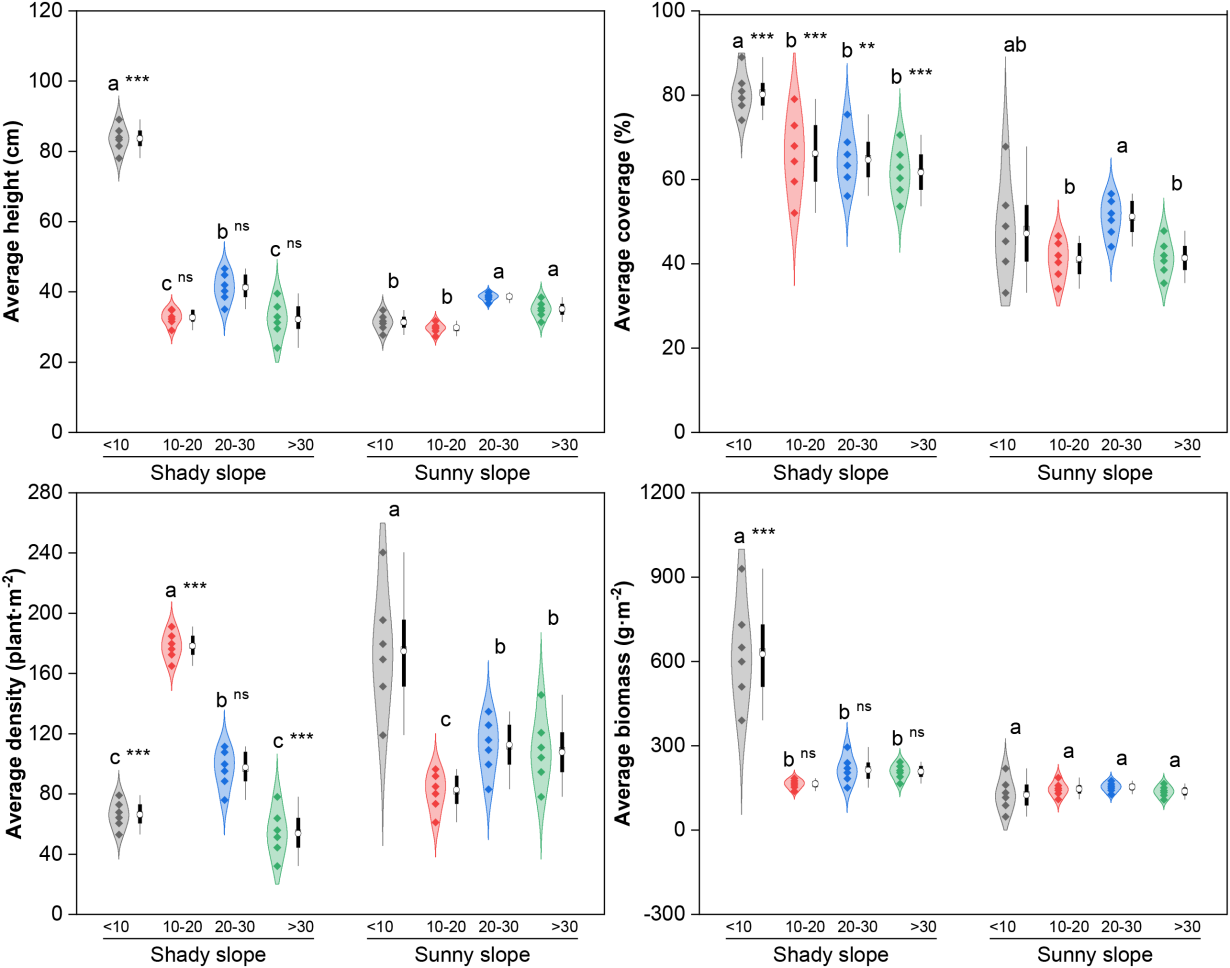
Effects of slope aspect and year of abandonment on the quantitative characteristics of the grassland plant community. In the combined plot, the violin plots represent the density estimation curves of the data, with the internal points representing the raw data; the boxes of the box plots represent the interquartile range (IQR) of the data, the square and circles on the box represent the average and median, respectively, and the whiskers indicate the range of nonoutlier values. On the basis of the results of *post hoc* pairwise comparisons from generalized linear mixed models (GLMMs), different lowercase letters indicate significant differences in the indices among different abandonment stages for the same slope aspect, with the values decreasing in the order of the lowercase letters. Additionally, ns, ** and *** represent nonsignificant and significant differences in the indices between the shady and sunny slopes for the same abandonment stage, corresponding to *P* < 0.01 and *P* < 0.001, respectively.

As shown in **Figure 1**, the height, coverage, and biomass of the shady slope communities decreased with increasing abandonment years, with significantly greater values in the initial stage (*P* < 0.05). In contrast, sunny slope communities had increasing height (significantly greater in the middle–late and late stages, *P* < 0.05), slightly decreasing coverage (significantly greater in the middle–late stage, *P* < 0.05), and stable biomass (*P*>0.05). Compared with the sunny slopes, the shady slopes had higher height and biomass only in the initial stage but higher coverage across all stages (*P* < 0.05). Shady slope density showed a “first increasing but then decreasing” trend (peak in the middle stage, *P* < 0.05), in contrast to that of sunny slopes; density dominance between slopes reversed in the middle stage (*P* < 0.05).

### 3.2 Response of species number, biomass and density of different functional groups in abandoned grassland communities to slope aspect and year of abandonment

The GLMM results indicated that, except for a few indicators, slope aspect, abandonment year, and their interaction had significant effects on the species number, biomass, and density of different functional groups in grassland communities (*P* < 0.05; **Supplementary Table 3**). Also, although the random effects of quadrat were significant, its proportion of its explanation for model variation for every functional group trait was quite low (0.47~1.02%, *P* < 0.05; **Supplementary Table 3**). Therefore, in the subsequent analysis, the influence of fixed effects is mainly considered.

**Figure 2:**
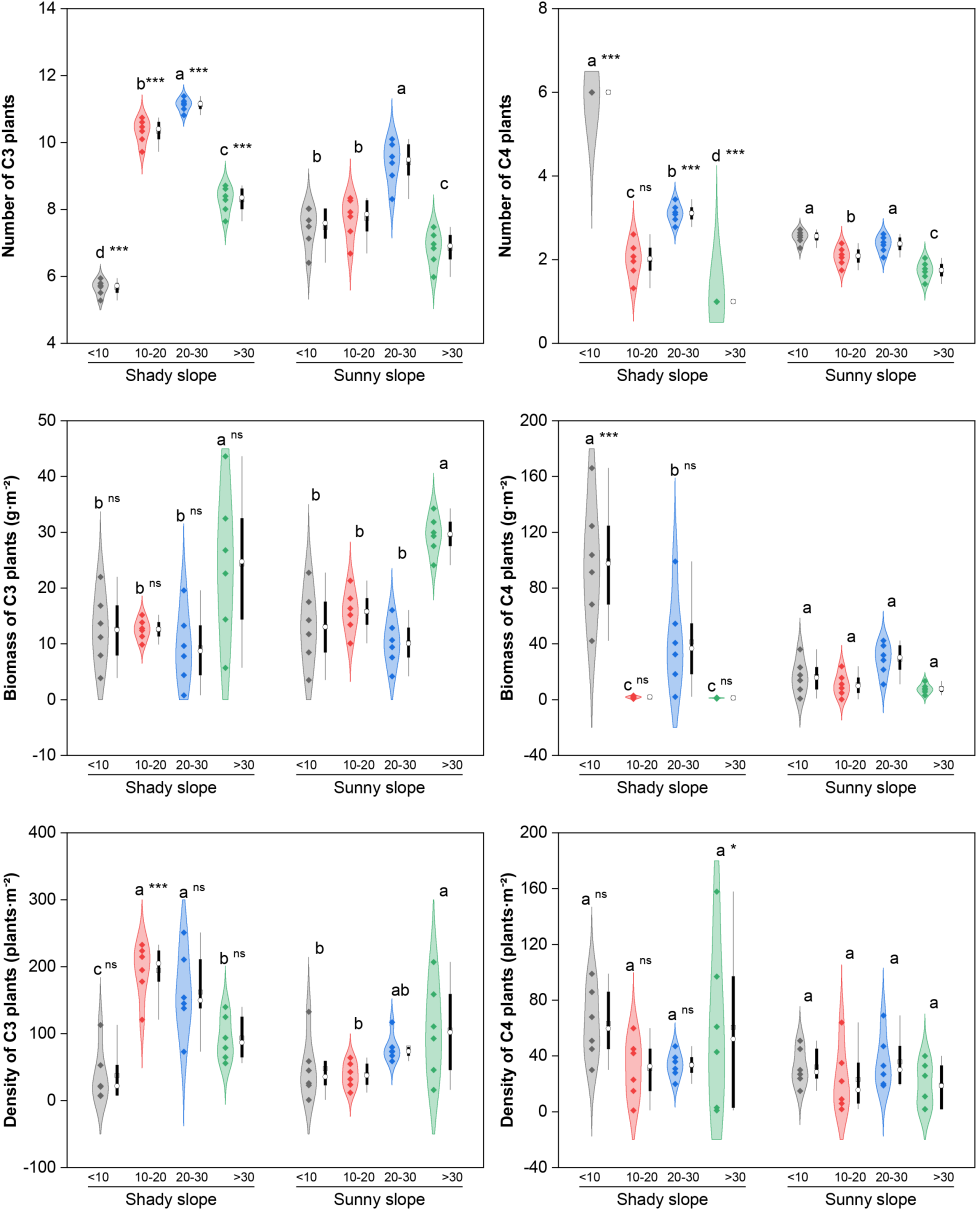
Effects of slope aspect and year of abandonment on the species number, biomass and density of plants with different photosynthetic pathways in the grassland plant community. In the combined plot, the violin plots represent the density estimation curves of the data, with the internal points representing the raw data; the boxes of the box plots represent the interquartile range (IQR) of the data, the square and circles on the box represent the average and median, respectively, and the whiskers indicate the range of nonoutlier values. On the basis of the results of *post hoc* pairwise comparisons from generalized linear mixed models (GLMMs), different lowercase letters indicate significant differences in the indices among different abandonment stages for the same slope aspect, with the values decreasing in the order of the lowercase letters. Additionally, ns, * and *** represent nonsignificant and significant differences in the indices between the shady and sunny slopes for the same abandonment stage, corresponding to *P* > 0.05, *P* < 0.05 and *P* < 0.001, respectively.

**Figure 3:**
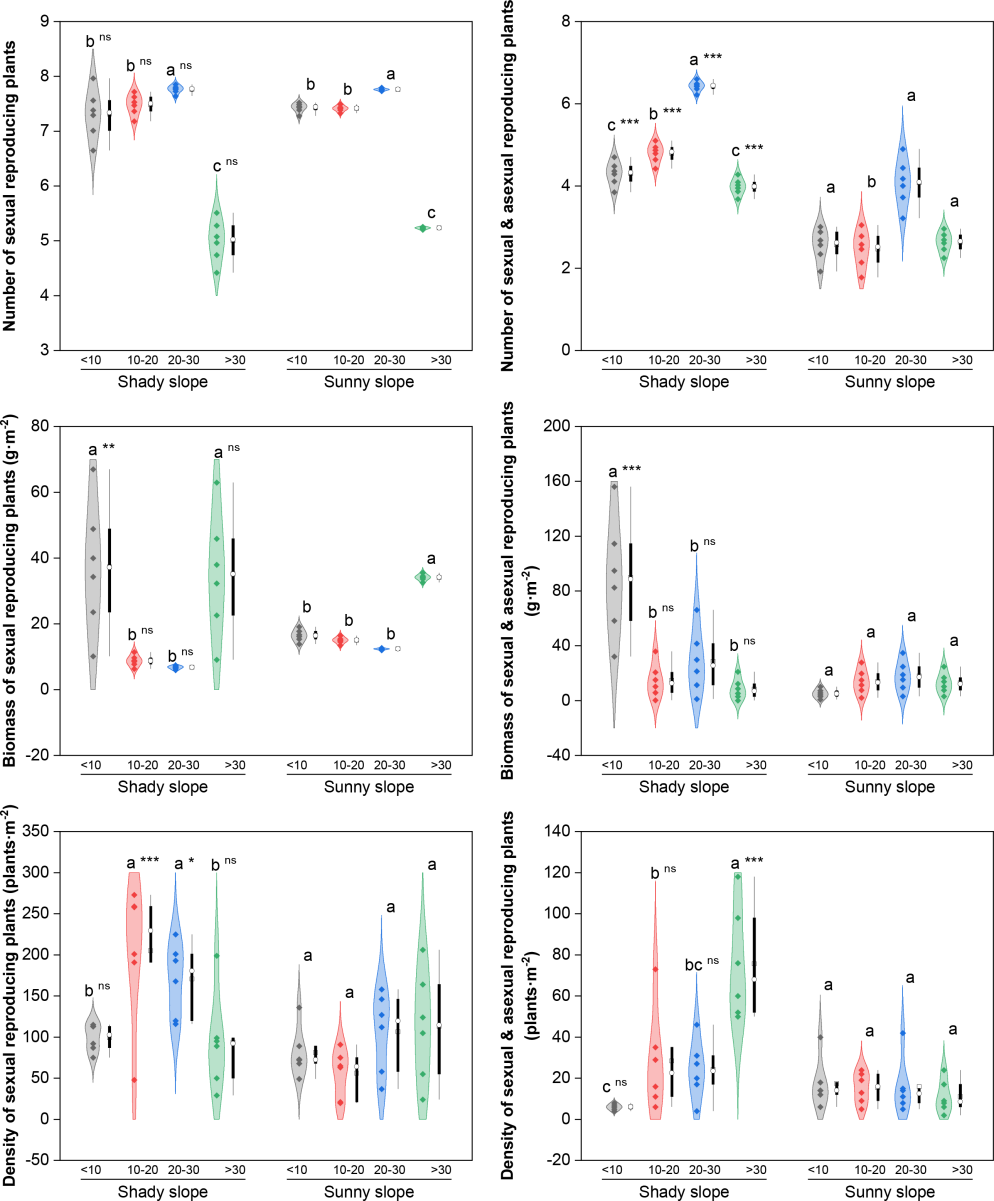
Effects of slope aspect and year of abandonment on the species number, biomass and density of plants with different reproductive modes in the grassland plant community. In the combined plot, the violin plots represent the density estimation curves of the data, with the internal points representing the raw data; the boxes of the box plots represent the interquartile range (IQR) of the data, the square and circles on the box represent the average and median, respectively, and the whiskers indicate the range of nonoutlier values. On the basis of the results of *post hoc* pairwise comparisons from generalized linear mixed models (GLMMs), different lowercase letters indicate significant differences in the indices among different abandonment stages for the same slope aspect, with the values decreasing in the order of the lowercase letters. Additionally, ns, * and *** represent nonsignificant and significant differences in the indices between the shady and sunny slopes for the same abandonment stage, corresponding to *P* > 0.05, *P* < 0.05, and *P* < 0.001, respectively.

**Figure 4:**
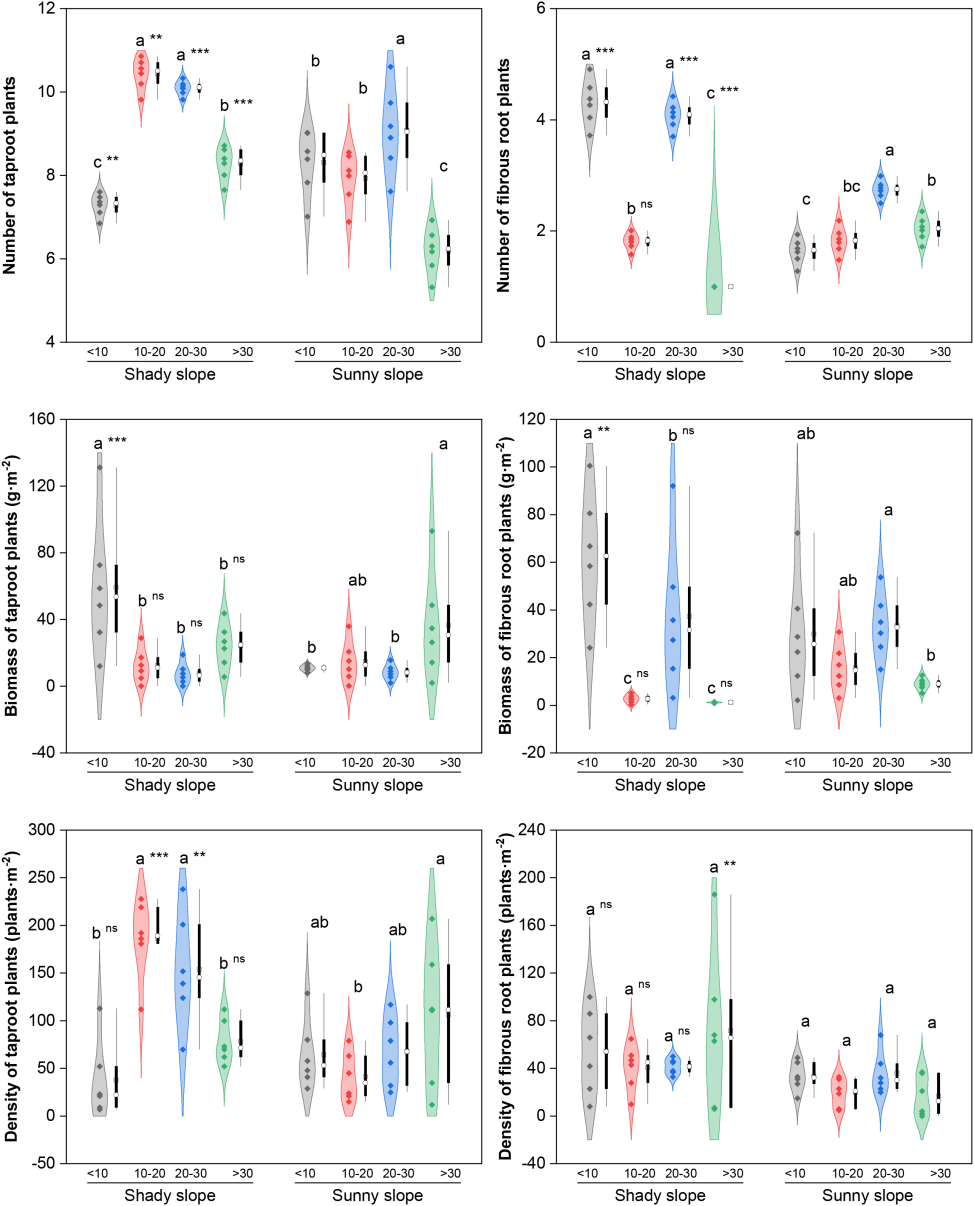
Effects of slope aspect and year of abandonment on the species number, biomass and density of plants with different root types in the grassland plant community. In the combined plot, the violin plots represent the density estimation curves of the data, with the internal points representing the raw data; the boxes of the box plots represent the interquartile range (IQR) of the data, the square and circles on the box represent the average and median, respectively, and the whiskers indicate the range of nonoutlier values. On the basis of the results of *post hoc* pairwise comparisons from generalized linear mixed models (GLMMs), different lowercase letters indicate significant differences in the indices among different abandonment stages for the same slope aspect, with the values decreasing in the order of the lowercase letters. Additionally, ns, *, ** and *** represent nonsignificant and significant differences in the indices between the shady and sunny slopes for the same abandonment stage, corresponding to *P* > 0.05, *P* < 0.05, *P* < 0.01 and *P* < 0.001, respectively.

**Figure 5:**
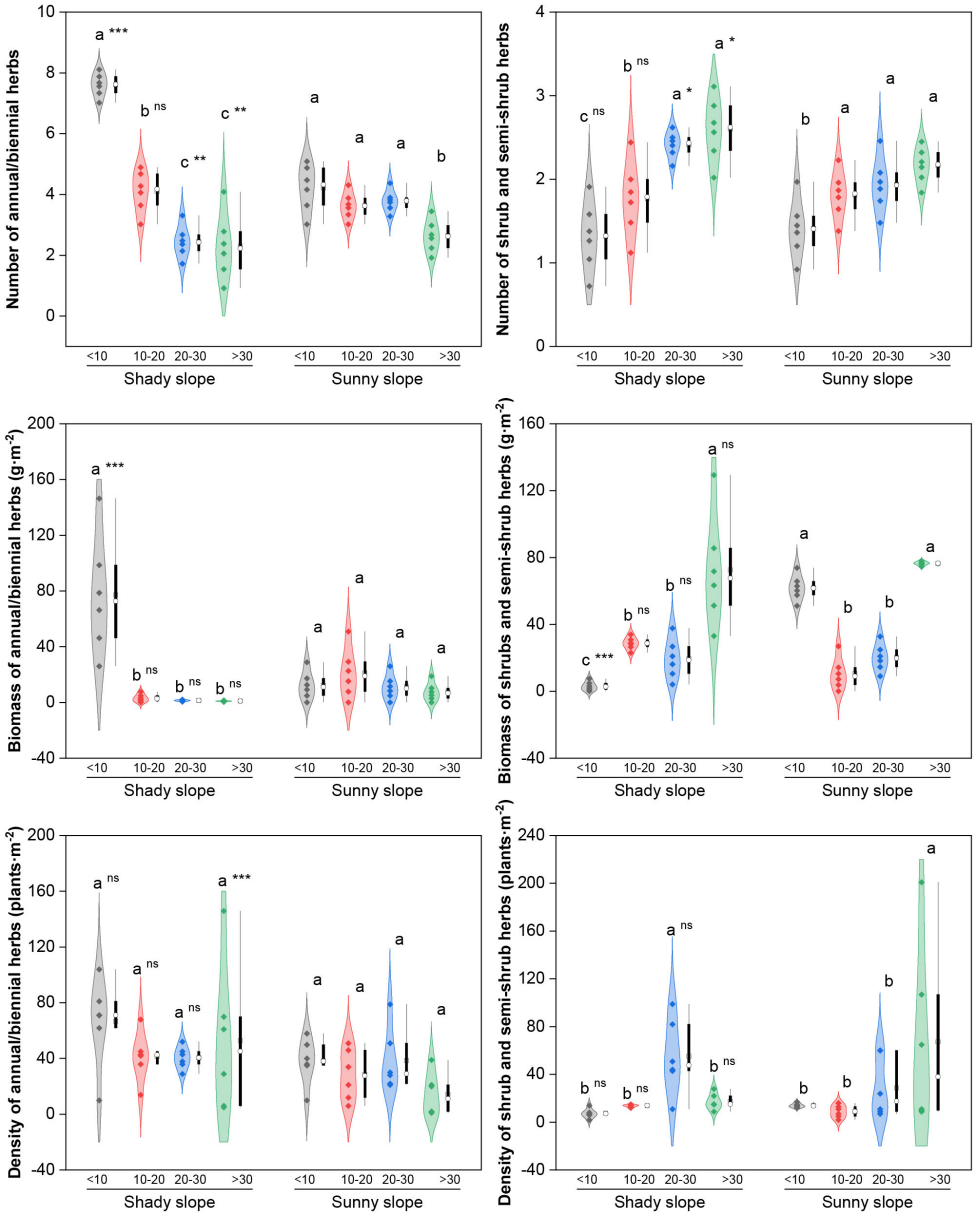
Effects of slope aspect and year of abandonment on the species number, biomass and density of plants with different growth forms in the grassland plant community. In the combined plot, the violin plots represent the density estimation curves of the data, with the internal points representing the raw data; the boxes of the box plots represent the interquartile range (IQR) of the data, the square and circles on the box represent the average and median, respectively, and the whiskers indicate the range of nonoutlier values. On the basis of the results of *post hoc* pairwise comparisons from generalized linear mixed models (GLMMs), different lowercase letters indicate significant differences in the indices among different abandonment stages for the same slope aspect, with the values decreasing in the order of the lowercase letters. Additionally, ns, *, ** and *** represent nonsignificant and significant differences in the indices between the shady and sunny slopes for the same abandonment stage, corresponding to *P* > 0.05, *P* < 0.05, *P* < 0.01 and *P* < 0.001, respectively.

The key response trends of each functional group to the two factors are summarized as follows, with detailed data shown in **Figures 2**-**5**. There were significant differences in the response of C3 and C4 plants to slope aspect and abandonment years (**Figure 2**). With respect to C3 plants, the number of species on both the shady and sunny slopes tended to first increase but then decrease, with the peak value occurring during the middle–late abandonment stage (*P* < 0.05), and the biomass significantly increased during the late abandonment stage (*P* < 0.05). The density of C3 plants showed a “first increasing but then decreasing” trend on the shady slopes but an increasing trend on the sunny slopes, and significant differences were observed in the corresponding key stages (*P* < 0.05). With respect to C4 plants, the number of species on both slope aspects tended to decrease, with significantly greater values in the initial abandonment stage than in the other stages (*P* < 0.05), and the density remained stable (*P*>0.05). The biomass of C4 plants on shady slopes tended to decrease (at peak in the initial stage, *P* < 0.05), whereas no significant change was observed on sunny slopes (*P*>0.05). Compared with sunny slopes, shady slopes had significantly greater C4-related indices during key abandonment stages (*P* < 0.05), whereas a significant difference in C3 plants between the two slope aspects was found only in terms of species number during the initial abandonment stage (*P* < 0.05).

Reproductive functional groups (sexually reproducing, SR; sexual and asexual reproducing, S&AR, **Figure 3**) showed similar trends in terms of species number, with a “first increase and then decrease” pattern and a peak value in the middle–late abandonment stage (*P* < 0.05). With respect to the SR plants, the biomass and density on the shady slopes fluctuated, with the highest values occurring during the initial/late and middle abandonment stages, respectively (*P* < 0.05); on the sunny slopes, the biomass increased significantly (peak in the late stage, *P* < 0.05), while the density remained stable (*P*>0.05). With respect to the S&AR plants, the biomass on the shady slopes decreased (peak in the initial stage, *P* < 0.05), and the density increased (peak in the late stage, *P* < 0.05), whereas no significant changes in biomass or density were detected on the sunny slopes (*P*>0.05). Compared with sunny slopes, shady slopes had significantly greater SR-related indices in the early abandonment stage and more S&AR species in all abandonment stages (all *P* < 0.05).

Root functional groups (taproots, TRs; fibrous roots, FRs) differed mainly in their response in biomass (**Figure 4**). With respect to TR plants, the number of species on both slope aspects tended to first increase but then decrease, with the peak value in the middle abandonment stage (*P* < 0.05); the biomass on the shady slopes decreased (the peak in the initial stage, *P* < 0.05), while the biomass and density on the sunny slopes increased significantly (the peak in the late stage, *P* < 0.05). With respect to the FR plants, both the number of species and the biomass on the shady slopes decreased, with the highest values occurring during the initial abandonment stage (*P* < 0.05); however, the number of species on the sunny slopes fluctuated (the peak in the middle–late stage, *P* < 0.05), and the density remained stable on both slope aspects (*P*>0.05). Compared with sunny slopes, shady slopes had significantly higher TR and FR-related indices during key abandonment stages (*P* < 0.05).

Herbaceous and shrubby functional groups (perennial forbs, PFs; perennial grasses, PGs; annual/biennial herbs, ABHs; and shrubs and semishrub herbs, S&SHs) showed varied response trends (**Figure 5**). PFs and PGs had consistent trends in terms of species number and biomass, both of which showed a “first increasing then decreasing” pattern, with peak values in the middle–late abandonment stage (*P* < 0.05). For ABHs, the species number and biomass on shady slopes decreased (peaks in the initial stage, *P* < 0.05), whereas the species number on sunny slopes fluctuated significantly (*P* < 0.05). The number of S&SH species increased on both slope aspects, with significantly higher values in the middle–late and late abandonment stages (*P* < 0.05). Significant differences in the indices of these functional groups were observed between the two slope aspects during key abandonment stages (*P* < 0.05).

### 3.3 Species diversity and functional diversity of abandoned grassland communities on different slope aspects

The GLMM results revealed significant effects of slope aspect, abandonment year, and their interaction on all diversity indices, while the random effects of quadrat on the diversity indicators were significant as well (*P* < 0.05; **Supplementary Table 4**). However, considering the quite low explanation for model variation for the aforementioned indicators (0.57~0.78%), only the influences of fixed effects were considered.

**Figure 6:**
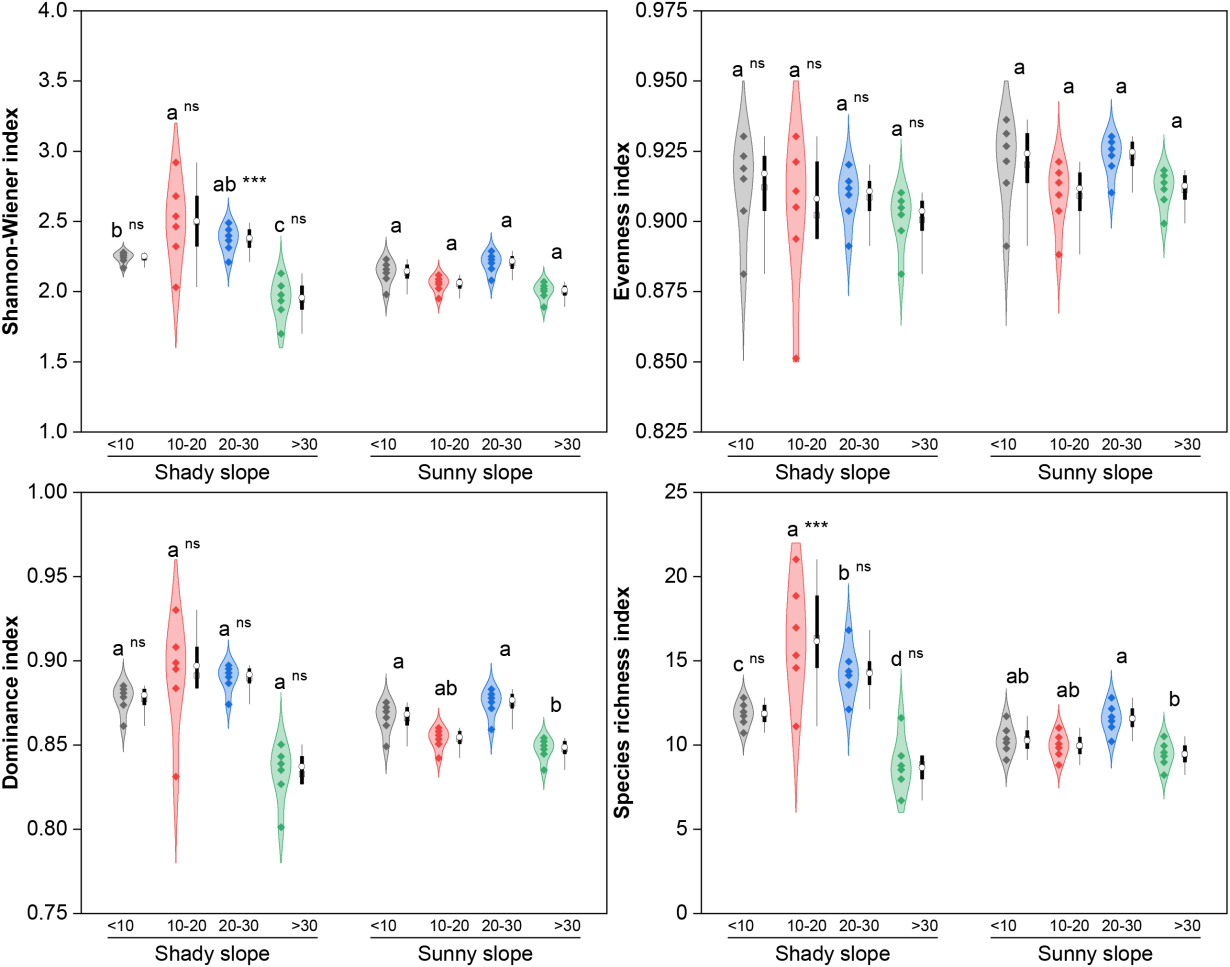
Effects of slope aspect and year of abandonment on the α diversity of the grassland plant community. In the combined plot, the violin plots represent the density estimation curves of the data, with the internal points representing the raw data; the boxes of the box plots represent the interquartile range (IQR) of the data, the square and circles on the box represent the average and median, respectively, and the whiskers indicate the range of nonoutlier values. On the basis of the results of *post hoc* pairwise comparisons from generalized linear mixed models (GLMMs), different lowercase letters indicate significant differences in the indices among different abandonment stages for the same slope aspect, with the values decreasing in the order of the lowercase letters. Additionally, ns and *** represent nonsignificant and significant differences in the indices between the shady and sunny slopes for the same abandonment stage, corresponding to *P* > 0.05 and *P* < 0.001, respectively.

**Figure 7:**
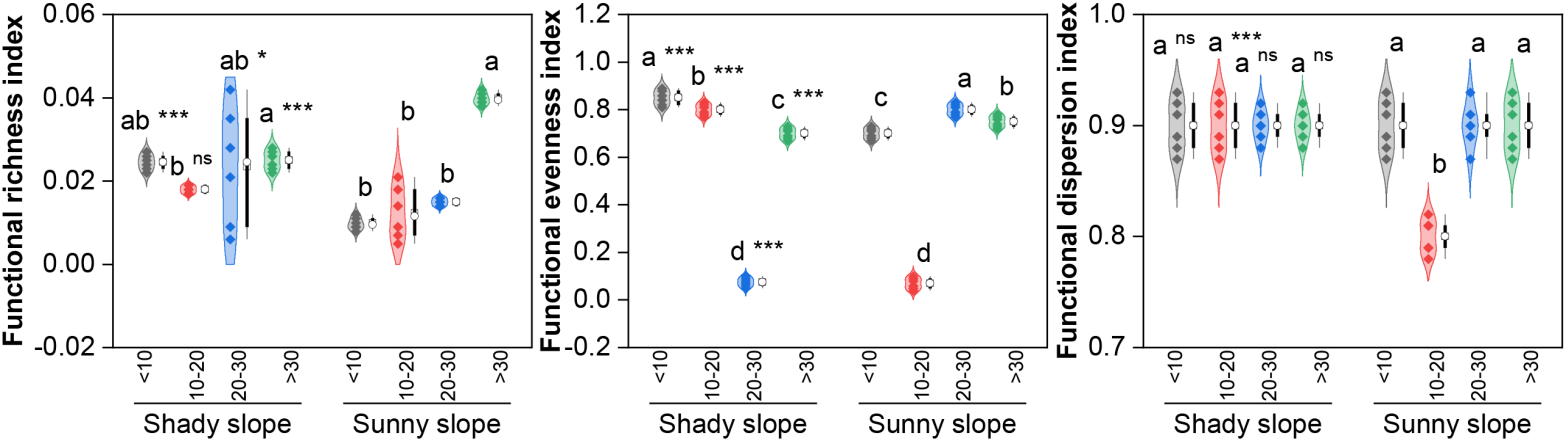
Effects of slope aspect and year of abandonment on the functional diversity of the grassland plant community. In the combined plot, the violin plots represent the density estimation curves of the data, with the internal points representing the raw data; the boxes of the box plots represent the interquartile range (IQR) of the data, the square and circles on the box represent the average and median, respectively, and the whiskers indicate the range of nonoutlier values. On the basis of the results of *post hoc* pairwise comparisons from generalized linear mixed models (GLMMs), different lowercase letters indicate significant differences in the indices among different abandonment stages for the same slope aspect, with the values decreasing in the order of the lowercase letters. Additionally, ns, * and *** represent nonsignificant and significant differences in the indices between the shady and sunny slopes for the same abandonment stage, corresponding to *P* > 0.05, *P* < 0.05 and *P* < 0.001, respectively.

With respect to species diversity (**Figure 6**), the Shannon–Wiener index and species richness of the shady slope first increased but then decreased (peak in the middle stage, *P* < 0.05), with stable evenness and dominance (*P*>0.05). Sunny slopes had stable Shannon–Wiener indices and evenness (*P*>0.05) and fluctuating dominance (higher in the initial/mid-late stages) and species richness (higher in the middle-late stage than in the late stage; *P* < 0.05). The Shannon–Wiener index (middle-late stage) and species richness (middle stage) were greater on the shady slopes than on the sunny slopes (*P* < 0.05), with no differences in the other stages (*P*>0.05). With respect to functional diversity (**Figure 7**), shady slopes had “first decrease but then increase” FRic and FEve (FRic higher in the late stage than in the middle stage and FEve higher in the initial stage, *P* < 0.05), with a stable FDis (*P*>0.05). Sunny slopes had increasing FRic (higher in the late stage, *P* < 0.05) and “first decrease then increase” FEve and FDis (lower in the middle stage, *P* < 0.05). Slope differences varied by stage: shady slopes had higher FRic (initial/middle-late stages, lower in late stage), higher FEve (initial/middle stages, lower in later stages), and higher FDis only in the middle stage (all *P* < 0.05).

## 4 Discussion and conclusions

Consistent with our hypothesis, slope aspect acts as the primary environmental filter that determines the initial habitat and successional trajectory, and significantly influences the temporal dynamics of plant communities in terms of structure, functional group characteristics, and diversity along the chronosequence of abandoned grassland.

### 4.1 Differentiated successional dynamics of plant community structure with increasing year of abandonment between slope aspects

In the initial stage of abandonment, the average height, coverage, and biomass of communities on shady slopes were higher than those on sunny slopes. This was because shady slopes had higher soil water content (on average 13% higher than sunny slopes; unpublished data) and lower evaporation rates, which facilitated plant colonization and growth (Gong and Gong, 2024; Wang et al., 2025; Xiong et al., 2026). Meanwhile, community density was higher on sunny slopes, driven by the rapid colonization of annual and biennial herbs (Zhang et al., 2021). With increasing year of abandonment, however, intensified inter- and intraspecific competition due to persistent water limitation gradually reduced plant density to its minimum on sunny slopes. In contrast, the relatively sufficient resources on shady slopes supported the highest plant density during the same period (Yirdaw et al., 2015). As abandonment continued further, shrubs and subshrubs with deep roots and efficient water-use strategies gradually dominated communities on sunny slopes, leading to a recovery in density. At the same time, increased canopy closure and light limitation intensified competition and reduced density on shady slopes (Che et al., 2022). These density dynamics reflect shifts in the relative importance of environmental filtering and species interactions along successional and resource gradients (Turner and Schweitzer, 2024). Regarding community height, soil water content on sunny slopes decreased by 25–30% compared with the initial stage (unpublished data) as abandonment progressed, imposing increasingly severe constraints on plant height growth and biomass accumulation in the later stages (Guo Z. et al., 2025). On shady slopes, the increase in community density likely favored taller plants with competitive advantages in light capture (Matsuo et al., 2024). Nevertheless, in the later stages, the increases in height and density on shady slopes were offset by reduced coverage, resulting in consistently low community biomass throughout abandonment.

### 4.2 Differentiated successional dynamics of plant functional group characteristics with increasing year of abandonment between slope aspects

When plants were grouped by photosynthetic pathway, root type, reproductive strategy, and growth form (Li et al., 2023), the enrichment or decline of specific functional strategies in grassland communities showed overall differences between slope aspects.

For photosynthetic functional groups, the density of C3 plants on shady slopes first increased and then decreased. This was related to the favorable combination of moderate light, temperature, and moisture in the initial abandonment stage that promoted plant colonization (Sun et al., 2019), and to suppressed enzyme activity caused by canopy closure and insufficient light in the middle and late stages (Wang et al., 2025). In contrast, high light intensity and long daylight hours on sunny slopes filtered for heliophilic C3 species (Li et al., 2018), leading to a continuous increase in their density. For C4 plants, density gradually declined on shady slopes due to canopy closure and light limitation. On sunny slopes, the physiological advantages of C4 plants—high water-use efficiency and low photorespiration (White et al., 2012; Yuan et al., 2025)—were offset by strong irradiance, persistent water loss, and unstable microclimates driven by large diurnal temperature fluctuations (Xu et al., 2022), resulting in non-significant changes in all indices throughout abandonment.

The divergent dynamics of reproductive functional groups essentially reflect differentialresponses of plant resource-allocation trade-offs during secondary succession following abandonment (Chen et al., 2025). The density of SR plants first increased and then decreased on shady slopes, but showed no significant change on sunny slopes. This difference arose because shady slopes experienced greater habitat fluctuation over long-term succession (Tian and Tian, 2026), leading to the decline of SR plants under competition from facultatively reproducing species. In contrast, the relatively stable habitat on sunny slopes resulted in non-significant changes over abandonment. For S&AR plants, biomass fluctuated slightly and density increased continuously on shady slopes, while no significant changes were observed on sunny slopes. However, their species number was consistently higher on shady slopes, likely because clonal integration combined with genetic recombination via sexual reproduction confers advantages in space occupation and resource consolidation (Mi et al., 2023; Wang et al., 2018), leading to directional selection in fluctuating environments.

For root functional groups, TR plants showed continuously decreasing biomass and a unimodal density pattern (increase then decrease) on shady slopes, whereas both biomass and density fluctuated and increased on sunny slopes. This is consistent with the advantage of deep roots in accessing deep soil water in the semi-arid Loess Hilly Region (Gao et al., 2016), representing directional selection for deep-rooted traits under persistent soil water deficit on sunny slopes. The species number of FR plants fluctuated and decreased on shady slopes, but showed a unimodal pattern (increase then decrease) on sunny slopes. This transition may be related to the progressive limitation of certain soil nutrients in the mid−late stage on sunny slopes (soil C and N contents decreased by 15–30% compared with the initial stage; unpublished data) Xue et al. (2022). Fibrous roots benefit the uptake of limited shallow nutrients in the early stage (Jiang et al., 2022) but become less competitive for deep nutrients in later stages, leading to decline. Their reduction on shady slopes may result from competitive exclusion by TR species.

Consistent with previous studies (Liu et al., 2023), ABHs generally declined across both slope aspects. However, their species number and biomass decreased continuously on shady slopes, with a faster rate than on sunny slopes. PG showed a unimodal trajectory (increase then decrease) on both slope aspects and became dominant in the mid−late stage. PGs had higher biomass on shady slopes, while shrubs and subshrubs increased in dominance on sunny slopes in later stages. This supports the theory that competitive species dominate under moderate resource availability, and stress-tolerant species dominate under resource scarcity (Pękalski and Wang, 2021). S&SHs increased on both slope aspects, indicating that shrub encroachment has occurred in these grasslands. The biomass of S&SHs on sunny slopes showed a fluctuating trajectory (decrease then increase), differing from the stable trend on shady slopes.

Notably, community-level functional reorganization in this study was achieved via species turnover rather than intraspecific trait variation or phenotypic plasticity. The shifts between competitive and stress-tolerant strategies observed during succession were fundamentally driven by slope aspect-dependent changes in interspecific competition. In the early abandonment stage, competition on the resource-poor sunny slopes centered mainly on water and nutrients. On shady slopes where soil water limitation was weaker, early competition was milder, allowing higher plant density and biomass. As succession entered the middle stage, competition for light intensified. On shady slopes, interspecific competition became significant with increasing canopy closure, yet average community height showed an overall decreasing trend—markedly contrasting with the continuous increase in height on sunny slopes.

### 4.3 Differentiated successional dynamics of plant species and functional diversity with increasing year of abandonment between slope aspects

For diversity, species diversity of communities on both shady and sunny slopes peaked in the middle abandonment stage (**Figure 6**), consistent with the intermediate disturbance hypothesis or the mid-successional peak hypothesis (Connell, 1978). The difference was that shady slopes provided favorable soil water and nutrient conditions (soil water 15–25% higher than sunny slopes; SOC, total N, and available N 35–51% higher; unpublished data), supporting diverse ecological niches for species with different survival strategies (Han et al., 2023; Wang et al., 2022). During the middle abandonment stage, neither early colonizers nor late dominants completely excluded other species, allowing short-term coexistence (Bar et al., 2023; Wang et al., 2022). Over time, however, environmental filtering selected for a limited set of species adapted to long-term site conditions (Zhang and Dong, 2010), leading to reduced species diversity. In contrast, the initially harsh hydrothermal conditions on sunny slopes constrained species colonization, resulting in smaller fluctuations in diversity indices.

On the other hand, the slope aspect-dependent divergence in community functional diversity reflects integrated responses of functional group composition and trait characteristics. The initial decrease and subsequent increase in functional richness and evenness on shady slopes were highly coupled with the successional dynamics of dominant functional groups such as C3 and TR species (**Figures 2**-**7**). The continuous increase in functional richness on sunny slopes arose from the gradual enrichment of drought-tolerant functional groups characterized by C4 photosynthesis, deep taproots or dense fibrous roots, and conservative leaf traits (**Figures 2**-**7**). The mid-successional difference in functional divergence indicates that functional trait dispersion decreased significantly on sunny slopes during the middle abandonment stage. This may be because water and nutrient limitations at this stage allowed only a few species with drought tolerance and resource-use efficiency to persist, reducing functional trait dispersion. These assumptions require further validation. Notably, diversity differences between slope aspects gradually weakened with increasing year of abandonment, suggesting that the effects of succession on community composition may gradually override initial topographic heterogeneity. However, even in the late abandonment stage, significant differences remained between shady and sunny slopes in functional diversity and certain functional group compositions, highlighting the persistent role of slope aspect heterogeneity in long-term abandonment.

### 4.4 Synergistic regulation of grassland community dynamics by year of abandonment and its interaction with slope aspect

Although this study focused on the effects of slope aspect on grassland successional dynamics, it is clear that year of abandonment significantly affected quantitative traits and diversity of grassland communities within each slope aspect, and exerted widespread regulatory effects on species number, biomass, and density of each functional group. This confirms that changes in hydrothermal conditions, soil nutrient accumulation, and interspecific competition collectively drive the transition of grassland ecosystems from a rapid colonization stage to a stable competitive stage over time (Shi et al., 2023), leading to corresponding shifts in community structure, functional properties, and diversity. The mechanisms have been extensively analyzed in previous sections and are not repeated here. Notably, the interactive effects of slope aspect and year of abandonment were significant for most indices. The core mechanism lies in their two-way regulation: On the one hand, slope aspect determines the habitat constraint threshold and modulates the strength and direction of abandonment age effects—habitat differences (e.g., water and light limitation) between slope aspects lead to divergent rates of habitat change and intensities of species competition under the same year of abandonment, resulting in heterogeneous successional trajectories. On the other hand, as a dynamic temporal factor, year of abandonment drives soil nutrient accumulation, species turnover, and community structural iteration, which in turn adjust the strength and mode of environmental filtering imposed by slope aspect. Admittedly, this study was conducted based on our initial hypothesis, and further investigation into the interactive effects of year of abandonment and slope aspect is still needed in future research. It should be noted that this study inferred community successional trajectories using a space-for-time substitution approach. In future work, long-term monitoring and analysis of plots with clear historical backgrounds are required to further verify and expand our conclusions. In addition, the relative importance of topographic factors and abandonment time may vary among ecosystems and climatic regions. Cross-regional comparative studies will help to develop a more general successional theoretical framework.

## Data Availability

The raw data supporting the conclusions of this article will be made available by the authors, without undue reservation.
